# Postoperative parenteral glutamine supplementation improves the short-term outcomes in patients undergoing colorectal cancer surgery: A propensity score matching study

**DOI:** 10.3389/fnut.2023.1040893

**Published:** 2023-03-16

**Authors:** Gang Tang, Feng Pi, Yu-Hao Qiu, Zheng-Qiang Wei

**Affiliations:** Department of Gastrointestinal Surgery, The First Affiliated Hospital of Chongqing Medical University, Chongqing, China

**Keywords:** glutamine, colorectal cancer, postoperative complications, intestinal function, albumin, propensity score

## Abstract

**Introduction:**

The clinical utility of glutamine in patients undergoing colorectal cancer (CRC) surgery remains unclear. Therefore, we aimed to investigate the impact of postoperative treatment with glutamine on postoperative outcomes in patients undergoing CRC surgery.

**Methods:**

We included patients with CRC undergoing elective surgery between January 2014 and January 2021. Patients were divided into the glutamine and control groups. We retrospectively analyzed postoperative infections complications within 30 days and other outcomes using propensity score matching and performed between-group comparisons.

**Results:**

We included 1,004 patients who underwent CRC surgeries; among them, 660 received parenteral glutamine supplementation. After matching, there were 342 patients in each group. The overall incidence of postoperative complications was 14.9 and 36.8% in the glutamine and control groups, respectively, indicating that glutamine significantly reduced the incidence of postoperative complications [*p* < 0.001; risk ratio (RR) 0.41 [95% CI 0.30–0.54]]. Compared with the control group, the glutamine group had a significantly lower postoperative infection complications rate (10.5 vs. 28.9%; *p* < 0.001; RR 0.36 [95% CI 0.26–0.52]). Although there was no significant between-group difference in the time to first fluid diet (*p* = 0.052), the time to first defecation (*p* < 0.001), first exhaust (*p* < 0.001), and first solid diet (*p* < 0.001), as well as hospital stay (*p* < 0.001) were significantly shorter in the glutamine group than in the control group. Furthermore, glutamine supplementation significantly reduced the incidence of postoperative intestinal obstruction (*p* = 0.046). Moreover, glutamine supplementation alleviated the decrease in albumin (*p* < 0.001), total protein (*p* < 0.001), and prealbumin levels (*p* < 0.001).

**Conclusions:**

Taken together, postoperative parenteral glutamine supplementation can effectively reduce the incidence of postoperative complications, promote the recovery of intestinal function, and improve albumin levels in patients undergoing CRC surgery.

## 1. Introduction

Worldwide, colorectal cancer (CRC) is the third most common cancer and among the most common mortality causes due to gastrointestinal cancer. In 2021, there were more than 1,800,000 new cases of CRC worldwide (1). Changes in diet and lifestyle have contributed to the incidence of CRC (2). Surgery has become the main treatment for CRC (3). Although there has been a gradual reduction in postoperative complications with the progress of perioperative nursing and technology, the incidence of complications after CRC surgery remains as high as 35% (4). Mechanical bowel preparation before CRC surgery can disrupt intestinal barrier function; further, potential intraoperative bacterial contamination may increase the risk of postoperative infectious complications (5, 6). Postoperative complications prolong hospital stay, increase hospitalization costs, and negatively affect the long-term prognosis of patients (7, 8). A recent meta-analysis reported that postoperative complications increased the risk of recurrence and decreased overall survival among patients with non-metastatic CRC (9). Therefore, reducing postoperative complications is crucial for improving the prognosis of patients.

Recent studies have demonstrated that immunonutritional therapy can reduce postoperative complications by regulating immune function in patients with CRC (8). Glutamine, which is crucially involved in immunonutrition, can regulate inflammatory response and immune balance, maintain the intestinal mucosal barrier, reduce intestinal damage, and reduce intestinal microbiota translocation (5, 8, 10). Accordingly, glutamine may provide a potential strategy for preventing postoperative complications. Additionally, a few studies have demonstrated that glutamine supplementation can reduce the length of hospital stay (5, 11).

However, there remains insufficient evidence for supporting the routine perioperative use of glutamine in patients with CRC. A recent meta-analysis recommended large-scale studies to evaluate the effect of perioperative glutamine supplementation in patients undergoing CRC surgery (8). Accordingly, we used propensity score matching (PSM) to investigate the effect of postoperative glutamine supplementation on postoperative complications and recovery in patients undergoing CRC surgery.

## 2. Methods

### 2.1. Study population

This retrospective cohort study was conducted between January 2014 and January 2021. This study was ethically approved by the Institutional Ethics Committee of the First Affiliated Hospital of Chongqing Medical University. All the patients provided informed consent.

We included patients who underwent elective surgery for primary CRC with and without glutamine supplementation (20% alanyl-glutamine 50–100 mL daily) for ≥5 days from the day of surgery. We excluded cases without primary anastomosis; cases with multiple primary cancers, a history of treatment for other abdominal or pelvic malignancy, or multi-visceral resections; patients with hepatic or renal failures; and emergency cases.

Standardized laparoscopic colorectal cancer and robotic surgery were adopted in our hospital, all of which were performed by four surgeons with an experience of more than 100 laparoscopic colorectal cancer operations. We collected information regarding baseline characteristics, intraoperative details, and postoperative recovery from the electronic medical record system. Tumor staging was determined based on American Joint Committee on Cancer, 8th edition (12). Serum prealbumin, total protein, and albumin levels on the day before surgery and 5 days after surgery were collected.

### 2.2. Primary and secondary endpoints

The primary endpoints were postoperative infection complications within 30 postoperative days, including anastomotic leakage, intra-abdominal infection (excluding anastomotic leakage), wound infection, pneumonia, and urinary infection. The secondary endpoints included serum total protein and albumin levels; time to first exhaust, defecation, fluid diet, and solid diet; rate of postoperative complications, rate of reoperation within 30 postoperative days, postoperative length of hospital stay, and mortality.

### 2.3. Statistical analysis

The sample size was calculated based on the primary endpoint. A previous study (5) reports that the incidence of infectious complications is 42%; accordingly, assuming an incidence rate of 11% in the glutamine group, ≥31 patients were expected to be required in each group to achieve 80% power with a two-tailed *p* value <0.05. Eligible participants were divided into the glutamine and control groups based on whether they received intravenous glutamine supplementation. Between-group comparisons of categorical and continuous variables were performed using the chi-square test and Mann–Whitney U test (Kolmogorov–Smirnov tests showed that the data in this study were non-normally distributed), respectively. Dichotomous variables were described using percentages, while continuous variables by median (interquartile range: 25–75 percentile). To reduce potential confounders resulting from between-group differences in baseline characteristics, we performed PSM analysis using patient demographics (male, age, BMI, tumor stage, and neoadjuvant therapy), comorbidities (chronic obstructive pulmonary disease, liver disorder, hypertension, diabetes mellitus, coronary artery disease, and American Society of Anesthesiologists Physical Status classification), malnutrition (preoperative prealbumin, preoperative total protein, and albumin), and surgical data (surgical approach, diverting stoma, duration of surgery, intraoperative blood loss, intraoperative transfusion, and conversion). Using the nearest neighbor matching algorithm, the matching ratio was 1:1. Calipers were set to 0.2 times the standard deviation of the logarithm of the estimated propensity score. All statistical analyses were performed using IBM SPSS version 26. Statistical significance was set at a two-sided *p* value <0.05.

## 3. Results

### 3.1. Baseline characteristics before and after propensity score matching

We included 1,004 consecutive patients undergoing elective surgery for CRC (38.6%, female; median age, 62 years). There were 660 and 344 patients in the glutamine and control groups, respectively. Before matching, there were a significant between-group difference in neoadjuvant therapy, duration of surgery, intraoperative blood loss, and intraoperative transfusion, but not in patient demographics (male, age, BMI, and tumor stage), comorbidities (chronic obstructive pulmonary disease, liver disorder, hypertension, diabetes mellitus, coronary artery disease, and American Society of Anesthesiologists physical status classification), malnutrition (preoperative prealbumin, preoperative total protein, and preoperative albumin), and surgical data (surgical approach, diverting stoma, and conversion; Table 1). After PSM, there were no significant differences in all covariates between the glutamine (*n* = 342) and control (*n* = 342) groups (Table 1).

**Table 1:** Baseline characteristics before and after propensity score matching.

	Group glutamine before PSM (*n* = 660)	Group Non- glutamine before PSM (*n* = 344)	*p* value ^b^	χ^2^	Group glutamine after PSM (*n* = 342)	Group non- glutamine after PSM (*n* = 342)	*p* value ^b^	χ^2^
Age (years)^a^	62 (54–69)	63 (53–69)	0.962	-	62 (54–69)	62.5 (53–69)	0.982	-
Gender (%)			0.278	1.176			0.430	0.622
Male	397 (60.2)	219 (63.7)			208 (60.8)	218 (63.7)		
Female	263 (39.8)	125 (36.3)			134 (39.2)	124 (36.3)		
BMI^a^	22.8 (20.7–24.6)	22.7 (20.6–24.5)	0.832	-	22.8 (20.8–24.4)	22.8 (20.7–24.5)	0.929	-
COPD (%)	38 (5.8)	23 (6.7)	0.559	0.342	19 (5.6)	23 (6.7)	0.524	0.406
Liver disorder (%)	20 (3)	9 (2.6)	0.710	0.138	10 (2.9)	9 (2.6)	0.816	0.054
Hypertension (%)	145 (22)	78 (22.7)	0.799	0.065	74 (21.6)	78 (22.8)	0.713	0.135
Diabetes mellitus (%)	76 (11.5)	37 (10.8)	0.718	0.131	42 (12.3)	37 (10.8)	0.550	0.358
Coronary artery disease (%)	56 (8.5)	22 (6.4)	0.240	1.378	28 (8.2)	22 (6.4)	0.378	0.777
ASA grade (%)			0.436	1.662			0.485	1.445
1	9 (1.4)	8 (2.3)			5 (1.5)	8 (2.3)		
2	485 (73.5)	244 (70.9)			254 (74.3)	242 (70.8)		
3	166 (25.2)	92 (26.7)			83 (24.3)	92 (26.9)		
Neoadjuvant therapy received (%)	29 (4.4)	26 (7.6)	0.037	4.373	15 (4.4)	26 (7.6)	0.076	3.139
Treatment modality (%)			0.951	0.004			0.533	0.389
Robotic/ Laparoscopy	621 (94.1)	324 (94.2)			318 (93)	322 (94.2)		
Conventional open	39 (5.9)	20 (5.8)			24 (7)	20 (5.8)		
Diverting stoma (%)	35 (5.3)	24 (7)	0.285	1.145	20 (5.8)	24 (7)	0.533	0.389
Duration of surgery (min)^a^	194.5 (150–240)	200 (160–250)	0.036	-	190 (150.8–245)	200 (160–250)	0.071	-
Intraoperative blood loss (ml)^a^	50 (20–100)	50 (20–100)	0.047	-	50 (20–100)	50 (20–100)	0.171	-
Transfusion (%)	7 (1.1)	12 (3.5)	0.007	7.179	4 (1.2)	10 (2.9)	0.105	2.625
Conversion (%)	5 (0.8)	3 (0.9)	0.846	0.038	2 (0.6)	3 (0.9)	0.654	0.201
Type of operation			0.131	7.093			0.546	3.072
Right hemicolectomy (%)	160 (24.2)	73 (21.2)			81 (23.7)	73 (21.3)		
Transverse colectomy	9 (1.4)	4 (1.2)			5 (1.5)	4 (1.2)		
Left hemicolectomy	36 (5.5)	13 (3.8)			16 (4.7)	13 (3.8)		
Sigmoid colectomy	110 (16.7)	45 (13.1)			55 (16.1)	45 (13.2)		
Anterior resection	345 (52.3)	209 (60.8)			185 (54.1)	207 (60.5)		
Year			0.192	8.683			0.279	7.472
2014	59 (8.9)	39 (11.3)			30 (8.8)	39 (11.4)		
2015	87 (13.2)	32 (9.3)			44 (12.9)	32 (9.4)		
2016	74 (11.2)	45 (13.1)			39 (11.4)	45 (13.2)		
2017	111 (16.8)	51 (14.8)			48 (14.0)	51 (14.9)		
2018	96 (14.5)	65 (18.9)			50 (14.6)	65 (19.0)		
2019	131 (19.8)	61 (17.7)			70 (20.5)	61 (17.8)		
2020^c^	102 (15.5)	51 (14.8)			61 (17.8)	49 (14.3)		
Preoperative prealbumin (mg/L)^a^	213.5 (188–234)	214 (188–237)	0.803	-	215 (186–237)	214 (188–238)	0.915	-
Preoperative total protein (g/L)^a^	68 (63–72)	68 (64–73)	0.125	-	69 (63–72)	68 (64–73)	0.438	-
Preoperative albumin (g/L)^a^	41 (38–44)	41 (37–44)	0.661	-	41 (37.8–45)	41 (37–44)	0.825	-
UICC stage (%)			0.288	2.487			0.731	0.627
I	128 (19.4)	71 (20.6)			72 (21.1)	70 (20.5)		
II	286 (43.3)	162 (47.1)			151 (44.2)	161 (47.1)		
III	246 (37.3)	111 (32.3)			119 (34.8)	111 (32.5)		

Values in parentheses are percentages unless indicated otherwise.

a Values are median (interquartile range: 25–75th percentile).

b Statistical analyses were performed using the chi-square test or Mann–Whitney U test;

c Including January 2021.

ASA, American Society of anesthesiologists physical status classification; BMI, body mass index; COPD, chronic obstructive pulmonary disease; and PSM, propensity score matching.

### 3.2. Postoperative short-term outcomes before and after propensity score matching

Before matching, the rate of postoperative complications was significantly lower in the glutamine group than in the control group [15.9 vs. 36.6%, respectively; *p* < 0.001; risk ratio (RR) 0.43 [95% CI 0.35–0.54]; Table 2]. After PSM, the overall incidence of postoperative complications in the glutamine and control groups was 14.9 and 36.8%, respectively (*p* < 0.001; RR 0.41 [95% CI 0.30–0.54]). The glutamine group had a significantly lower rate of postoperative infections than the control group (10.5 vs. 28.9%, respectively; *p* < 0.001; RR 0.36 [95% CI 0.26–0.52]). There was no significant between-group difference in the rate of wound infection (*p* = 0.105), urinary infection (*p* = 0.101), and bleeding at anastomotic site (*p* = 0.412); however, the glutamine group had a significantly lower rate of anastomotic leakage (*p* = 0.043), pulmonary tract infection (*p* = 0.007), and intraabdominal infection (*p* < 0.001) than the control group (Table 2).

**Table 2:** Operative outcomes before and after propensity score matching.

	Group glutamine before PSM (*n* = 660)	Group non- glutamine before PSM (*n* = 344)	*p* value^b^	χ^2^	Group glutamine after PSM (*n* = 342)	Group non- glutamine after PSM (*n* = 342)	*p* value^b^	χ^2^
Days to first flatus^a^	2 (1–3)	2 (2–3)	< 0.001	-	2 (1–3)	2 (2–3)	< 0.001	-
Days to first defecation^a^	3 (3–4)	4 (3–5)	< 0.001	-	3 (3–5)	4 (3–5)	< 0.001	-
Days to first fluid diet^a^	3 (2–3)	3 (2–3)	0.176	-	3 (2–3)	3 (2–3)	0.052	-
Days to first solid diet^a^	6 (5–7)	6 (5–7)	< 0.001	-	5 (4–7)	6 (5–7)	< 0.001	-
Reoperation (%)	3 (0.5)	2 (0.6)	0.786	0.073	2 (0.6)	2 (0.6)	1.00	0.000
Mortality (%)	2 (0.3)	2 (0.6)	0.506	0.442	2 (0.6)	2 (0.6)	1.00	0.000
Postoperative prealbumin (mg/L) ^a^	181 (156–198)	138 (107–164)	< 0.001	-	184 (159.8–199)	138 (107–164)	< 0.001	-
Postoperative total protein (g/L)^a^	61 (58–66)	59 (55–63)	< 0.001	-	61 (58–66)	59 (55–63)	< 0.001	-
Postoperative albumin (g/L)^a^	35 (32–38)	34 (31–37)	< 0.001	-	35 (32–38)	34 (31–37)	< 0.001	-
Hospital stay (days)^a^	8 (7–10)	9 (7–11)	< 0.001	-	8 (7–10)	9 (7–11)	< 0.001	-
Postoperative complications (%)	105 (15.9)	126 (36.6)	< 0.001	54.799	51 (14.9)	126 (36.8)	< 0.001	42.874
Urinary infection (%)	2 (0.3)	5 (1.5)	0.038	4.323	1 (0.3)	5 (1.5)	0.101	2.690
Pneumonia (%)	20 (3)	24 (7)	0.004	8.405	9 (2.6)	24 (7)	0.007	7.164
Ileus (%)	11 (1.7)	18 (5.2)	0.001	10.251	8 (2.3)	18 (5.3)	0.046	3.998
Wound infection (%)	13 (2.0)	10 (2.9)	0.346	0.888	4 (1.2)	10 (2.9)	0.105	2.625
Intraabdominal infection (%)	37 (5.6)	48 (14)	< 0.001	20.333	18 (5.3)	48 (14)	< 0.001	15.093
Anastomotic leakage (%)	8 (1.2)	12 (3.5)	0.014	6.001	4 (1.2)	12 (3.5)	0.043	4.096
Bleeding at anastomotic site (%)	4 (0.6)	4 (1.2)	0.346	0.887	2 (0.6)	4 (1.2)	0.412	0.673
Others (%)	10 (1.5)	5 (1.5)	0.939	0.006	5 (1.5)	5 (1.5)	1.000	0.000

Values in parentheses are percentages, unless indicated otherwise.

a Values are median (interquartile range: 25–75th percentile).

b Statistical analyses were performed using the chi-square test or Mann–Whitney U test.

PSM, propensity score matching.

Regarding postoperative intestinal function recovery, although there was no significant between-group difference in the time to first fluid diet (*p* = 0.052), the glutamine group showed a significantly shorter time to first exhaust (*p* < 0.001), first defecation (*p* < 0.001), and first solid diet (*p* < 0.001) than the control group. Additionally, glutamine supplementation significantly reduced the incidence of postoperative intestinal obstruction (*p* = 0.046). The median length of hospital stay was 8 and 9 days in the glutamine and control groups, respectively (*p* < 0.001). Moreover, the median hospitalization cost in the glutamine group (75871.5RMB) was comparable to that in the control group (84059.7RMB; *p* = 0.950). There was two death in the glutamine group and two deaths in the control group (Table 2).

The median postoperative total protein levels were 61 and 59 g/L in the glutamine and control groups, respectively (*p* < 0.001). Glutamine alleviated the decrease in perioperative albumin (*p* < 0.001) and prealbumin (*p* < 0.001) levels (Table 2). Details of Clavien-Dindo classification of postoperative complications are in Table 3.

**Table 3:** Clavien-Dindo classification of postoperative complication after propensity score matching.

	Group glutamine (*n* = 342)	Group non-glutamine (*n* = 342)	*p* value^a^	χ^2^
Clavien-Dindo classification			0.679	2.311
I	8 (2.3)	13 (3.8)		
II	31 (9.1)	87 (25.4)		
III	7 (2)	15 (4.4)		
IV	3 (0.9)	9 (2.6)		
V	2 (0.6)	2 (0.6)		-

Values in parentheses are percentages.

a Statistical analyses were performed using the chi-square test or Mann–Whitney U test.

## 4. Discussion

To our knowledge, this is the first large-scale study to explore the effects of glutamine on postoperative complications and recovery after CRC surgery. We found that glutamine supplementation could effectively reduce the incidence of postoperative complications; shorten the time to first exhaust, first defecation, and first solid diet; reduce the length of hospital stay; and improve serum prealbumin, total protein, and albumin levels. Postoperative complications negatively affect the short-term and long-term prognosis of patients with CRC. Colorectal surgery research has recently focused on the prevention of postoperative complications. Our findings provide current evidence regarding the prevention of postoperative complications and improvement of postoperative recovery in CRC surgery through glutamine supplementation.

Radical resection is the standard treatment for CRC (13). Immunonutrition therapy can effectively reduce the incidence and severity of postoperative complications in patients undergoing radical surgery for CRC (8). Glutamine is the preferred fuel for intestinal mucosal cells and immune cells; accordingly, it is crucially involved in regulating the body’s immune function and maintaining the integrity of the intestinal mucosal barrier (8, 14). Surgical trauma reduces plasma and intracellular glutamine pool levels, which impairs the normal immune function of T cells, the bactericidal function of neutrophils, the phagocytic activity of macrophages, and interleukin-1 production (15–17). The depletion of stored glutamine may cause postoperative complications, including infectious complications, abnormal immune function, increased intestinal permeability, poor wound healing, and even multiple organ failure (5). Low serum glutamine levels are associated with shortened survival of patients with CRC (18). Therefore, glutamine supplementation may be crucial for preventing postoperative complications. In our study, glutamine supplementation effectively reduced the incidence of postoperative complications. Several studies have demonstrated that glutamine supplementation can reduce postoperative complications. O’Riordain et al. (17) found that glutamine supplementation enhanced postoperative T lymphocyte immune function in patients undergoing colorectal surgery. In a study conducted by Cui et al. (11), patients with colon cancer received 0.5 g/kg glutamine 24 h before and 1 h after surgery and found that glutamine supplementation reduced the incidence of postoperative complications. Similarly, Oguz et al. reported that intravenous glutamine supplementation reduced postoperative complications (5). Additionally, the incidence of anastomotic leakage is as high as 3–20% and is related to increased postoperative morbidity, mortality, permanent stoma rate, and recurrence rate (13, 19–21). Accordingly, we focused on the important complication of an anastomotic leak. We found that glutamine supplementation effectively reduced the incidence of anastomotic leaks. Consistent with this finding, Yang et al. reported a significantly lower incidence of anastomotic leak in the glutamine group than in the control group (RR = 0.23, 95% CI: 0.09–0.61) (8). This could be attributed to several factors. On the one hand, glutamine can increase collagen synthesis, and thus accelerate intestinal mucosal healing and regeneration (13, 22). On the other hand, inflammation severity is among the important factors affecting the healing of intestinal anastomosis. Accordingly, glutamine can reduce inflammatory injury and oxidative stress as well as protect the healing of anastomosis (13).

In addition to reducing postoperative complications, glutamine may also promote postoperative recovery of gastrointestinal function. Glutamine can prevent intestinal mucosal atrophy and protect the intestinal mucosal barrier (17). Glutamine supplementation has been shown to prevent chemotherapy-induced diarrhea (23). Using animal experiments with dogs, Ohno et al. reported that glutamine improved intestinal obstruction after abdominal surgery (24). Additionally, Ohno et al. (25) reported that glutamine supplementation improved the decrease in plasma glutamine levels and gastrointestinal motility after gastrectomy. Our findings demonstrated that glutamine supplementation promoted intestinal function recovery, including shortening the time to first exhaust, first defecation, and first solid diet as well as reducing the incidence of postoperative ileus. Additionally, we found that glutamine reduced postoperative hospital stay, which is consistent with previous reports (5, 11, 26) and could be attributed to decreased complications and improved recovery of intestinal function. This in turn could have contributed to reduced hospitalization costs. Thus we observed that additional glutamine supplementation did not increase total hospitalization costs.

Hypoproteinemia is related to increased postoperative morbidity and prolonged hospital stay (27, 28). Major gastrointestinal surgery is often accompanied by a high inflammatory response, which impairs liver protein metabolism. Glutamine can increase hepatocyte synthesis and improve hepatic metabolism (29). Wu et al. reported that glutamine supplementation increased serum albumin levels in patients with gastric cancer undergoing radical surgery (29), which is consistent with our findings. However, while the differences in these indicators (total protein levels, albumin levels, and prealbumin levels) were statistically significant, whether these differences are of significant clinical value remains to be determined.

Our study has two strengths. On the one hand, it is the study with the largest sample size to examine the effects of glutamine on patients undergoing colorectal cancer surgery. On the other hand, we used PSM to balance potential confounding factors between the groups.

This study has several limitations. First, this was a single-center retrospective study and there may be potential confounding factors. Second, we retrospectively collected information regarding the time to first exhaust, defecation, fluid diet, and first solid diet from the electronic medical records, which could lead to potential bias. Prospective studies are warranted to confirm the benefits of glutamine supplementation on the recovery of bowel function after surgery for CRC. Then, we did not measure plasma glutamine levels before and after the intervention. Future studies should consider plasma glutamine levels. Finally, as the current guidelines do not clarify whether or not to supplement glutamine in patients undergoing colorectal cancer surgery, in this study, this decision was not based on clear criteria. This could have led to some selection bias. Given these limitations, prospective randomized controlled studies are needed to validate the benefit of glutamine supplements in patients undergoing colorectal cancer surgery.

In conclusion, we found that postoperative intravenous glutamine supplementation could effectively reduce the incidence of postoperative complications, promote the recovery of intestinal function, and improve albumin levels in patients undergoing CRC surgery.

## Data availability statement

The original contributions presented in the study are included in the article/supplementary material, further inquiries can be directed to the corresponding author.

## Ethics statement

The studies involving human participants were reviewed and approved by the Institutional Ethics Committee of the First Affiliated Hospital of Chongqing Medical University. The patients/participants provided their written informed consent to participate in this study.

## Author contributions

Z-QW, FP, and GT: conceptualization. FP, GT, and Y-HQ: data collection and analyses. GT and FP: writing—original draft preparation. Z-QW, FP, GT, and Y-HQ: writing—review and editing and had primary responsibility for the final content. All authors contributed to the article and approved the submitted version.

## Funding

This study was funded by Chongqing Key Diseases Research and Application Demonstration Program, No. 2019ZX003.

## Conflict of interest

The authors declare that the research was conducted in the absence of any commercial or financial relationships that could be construed as a potential conflict of interest.

## Publisher’s note

All claims expressed in this article are solely those of the authors and do not necessarily represent those of their affiliated organizations, or those of the publisher, the editors and the reviewers. Any product that may be evaluated in this article, or claim that may be made by its manufacturer, is not guaranteed or endorsed by the publisher.
